# Multi-center, randomized, placebo-controlled trial of nocturnal oxygen therapy in chronic obstructive pulmonary disease: a study protocol for the INOX trial

**DOI:** 10.1186/s12890-016-0343-9

**Published:** 2017-01-09

**Authors:** Yves Lacasse, Sarah Bernard, Frédéric Sériès, Van Hung Nguyen, Jean Bourbeau, Shawn Aaron, François Maltais, for the International Nocturnal Oxygen (INOX) Research Group, Marcel Mallet, Jean Bourbeau, Bruno Paradis, François Maltais, Richard Lecours, Pierre Larivée, Marc Baltzan, François Corbeil, Christine Drapeau, Guy Cournoyer, Shawn Aaron, Denis O’Donnell, Martha Shepertecky, Eric Wong, Jeremy Road, Paula Simão, Miguel Guimarães, Cristina Bárbara, Paula Pinto, Joaquim Moita, Cidália Rodrigues, João Munhá, Salete Valente, Carlos Javier Egea Santaolalla, Araceli Abad Fernández, Irene Cano, Javier Sayas Catalán, Cristóbal Esteban, Amaia Garcia-Loizaga, Jean-Claude Meurice, Alain Palot, Pascal Chanez, Jésus Gonzalez, Antoine Guerder

**Affiliations:** 1 Centre de recherche, Institut universitaire de cardiologie et de pneumologie de Québec (IUCPQ), 2725 Chemin Ste-Foy, Québec, Québec G1V 4G5 Canada; 2 Institut thoracique de Montréal, 3650 rue St-Urbain, Montréal, Québec H2X 2P4 Canada; 3 The Ottawa Hospital - General Campus, Mailbox 211, 501 Smyth Road, Ottawa, ON K1H 8L6 Canada

**Keywords:** COPD, Sleep, Oxygen therapy, Mortality, Randomized trial

## Abstract

**Background:**

Long-term oxygen therapy (LTOT) is the only component of the management of chronic obstructive pulmonary disease (COPD) that improves survival in patients with severe daytime hypoxemia. LTOT is usually provided by a stationary oxygen concentrator and is recommended to be used for at least 15–18 h a day. Several studies have demonstrated a deterioration in arterial blood gas pressures and oxygen saturation during sleep in patients with COPD, even in those not qualifying for LTOT. The suggestion has been made that the natural progression of COPD to its end stages of chronic pulmonary hypertension, severe hypoxemia, right heart failure, and death is dependent upon the severity of desaturation occurring during sleep. The primary objective of the International Nocturnal Oxygen (INOX) trial is to determine, in patients with COPD not qualifying for LTOT but who present significant nocturnal arterial oxygen desaturation, whether nocturnal oxygen provided for a period of 3 years decreases mortality or delay the prescription of LTOT.

**Methods:**

The INOX trial is a 3-year, multi-center, placebo-controlled, randomized trial of nocturnal oxygen therapy added to usual care. Eligible patients are those with a diagnosis of COPD supported by a history of past smoking and obstructive disease who fulfill our definition of significant nocturnal oxygen desaturation (i.e., ≥ 30% of the recording time with transcutaneous arterial oxygen saturation < 90% on either of two consecutive recordings). Patients allocated in the control group receive room air delivered by a concentrator modified to deliver 21% oxygen. The comparison is double blind. The primary outcome is a composite of mortality from all cause or requirement for LTOT. Secondary outcomes include quality of life and utility measures, costs from a societal perspective and compliance with oxygen therapy. The follow-up period is intended to last at least 3 years.

**Discussion:**

The benefits of LTOT have been demonstrated whereas those of nocturnal oxygen therapy alone have not. The INOX trial will likely determine whether supplemental oxygen during sleep is effective in reducing mortality, delaying the need for LTOT and improving health-related quality of life in patients with COPD who desaturate overnight.

**Trial registration:**

Current Controlled Trials ISRCTN50085100; ClinicalTrials.gov NCT01044628 (date of registration: January 6, 2010).

## Background

Chronic obstructive pulmonary disease (COPD) represents a major health issue worldwide. For instance, while 4% of Canadians aged 35 to 79 self-reported being diagnosed with COPD, direct measurements of lung function from the Canadian Health Measures Survey indicate that 13% of Canadians had a lung function score indicative of COPD. Among individuals aged > 40 with a smoking history of at least 20 pack-years visiting a primary care physician for any reason, one in five met spirometric criteria for COPD [1]. COPD represents the fourth leading cause of mortality in Canada [2]. Continuous (i.e., long-term) oxygen therapy (LTOT) is one of the few components of the management of COPD that improves survival and it is only indicated in patients with severe daytime hypoxemia [3, 4]. LTOT is usually provided by a stationary oxygen concentrator and should be used for at least 15 h a day [5].

Sleep-related non-apneic oxygen desaturation often occurs in patients not qualifying for LTOT [6–10] and is considered by many physicians as an indication for providing nocturnal oxygen therapy (NOT). This perceived indication stems from the suggestion that the natural progression of COPD to its end stages of severe hypoxemia, right heart failure, and death may be dependent upon the severity of desaturation occurring during sleep [11–13]. This attractive hypothesis is supported by the fact that hypoxemic episodes during sleep are accompanied by increases in pulmonary arterial pressure [14–16] and often by important cardiac arrhythmias[17, 18], both alleviated by nocturnal oxygen supplementation. Over the long run, NOT may halt the progression of long-standing cor pulmonale [4, 14] and may prolong survival [3].

Practice guidelines regarding the indications for NOT in COPD not qualifying for conventional LTOT are presently imprecise. Because of this, a number of these patients are currently treated with NOT [19] despite the fact that the clinical benefits of NOT have yet to be confirmed. Three randomized trials directly addressed the issue of the effectiveness of NOT in patients not qualifying for LTOT who desaturate overnight [20–22]. Two looked at the effect of NOT on survival at 2- to 3-year follow-up [21, 22]. Both trials were negative but were underpowered. Even their meta-analysis could not determine whether NOT improves survival, with a pooled mortality odds ratio of 0.97 and a wide 95% confidence interval (CI: 0.41–2.31) from which both detrimental and positive effects of NOT on survival could not be excluded [23]. We complemented this mortality meta-analyses by conducting a systematic review and meta-analysis of the composite outcome of mortality or requirement for LTOT (a surrogate marker of disease progression) and found no significant difference between the treated and the control groups (pooled odds ratio: 1.57 [95% CI: 0.75–3.26]; unpublished data).

Accordingly, the primary objective of the International Nocturnal Oxygen (INOX) trial is to determine, in patients with COPD not qualifying for LTOT who exhibit significant nocturnal arterial oxygen desaturation, whether NOT provided for a period of 3 years decreases mortality or the requirement for LTOT. Its secondary objectives are to examine whether NOT improves disease-specific quality of life and to calculate the incremental cost-effectiveness ratio of NOT.

## Methods/Design

### Study design

The INOX trial is a 3-year, multi-center, randomized, double-blind, placebo-controlled trial of NOT, with intention-to-treat analysis.

### Participants

The trial is currently conducted in 27 university-affiliated clinical sites in Canada, Portugal, Spain and France (Appendix).

#### Inclusion criteria

To be included in the trial, patients must fulfill all the following criteria:

- a diagnosis of COPD supported by a history of past smoking and obstructive disease: forced expiratory volume in 1 s (FEV1) < 70% predicted, FEV1/forced vital capacity (FVC) < 70% and a total lung capacity by body plethysmography > 80% predicted;
- stable COPD at study entry, as demonstrated by no acute exacerbation and no change in medications for at least 6 weeks before enrollment in the trial;
- non-smoker for at least 6 months before enrollment in the trial;
- fulfilling our definition of nocturnal oxygen desaturation (see below);
- ability to give informed consent.

#### Exclusion criteria

The exclusion criteria are the following:

- patients with severe hypoxemia fulfilling the usual criteria for LTOT at study entry [3]: PaO2 ≤ 55 mmHg; OR PaO2 ≤ 59 mmHg with clinical evidence of at least one of the following: (1) peripheral edema (cor pulmonale); (2) hematocrit ≥ 55%; (3) right ventricular hypertrophy (P pulmonale on ECG: 3 mm in leads II, III, aVf;
- patients with proven sleep apnea (defined by an apnea/hypopnea index of ≥ 15 events/hour [24]) or suspected sleep apnea on oximetry tracings;
- patients currently using NOT;
- patients with known left heart or congenital heart diseases, interstitial lung diseases, bronchiectasis as the main cause of obstructive disease, lung carcinoma, severe obesity (body mass index ≥ 40 kg/m^2^), or any other disease that could influence survival.

#### Operational definition of nocturnal desaturation

**Fig. 1:**
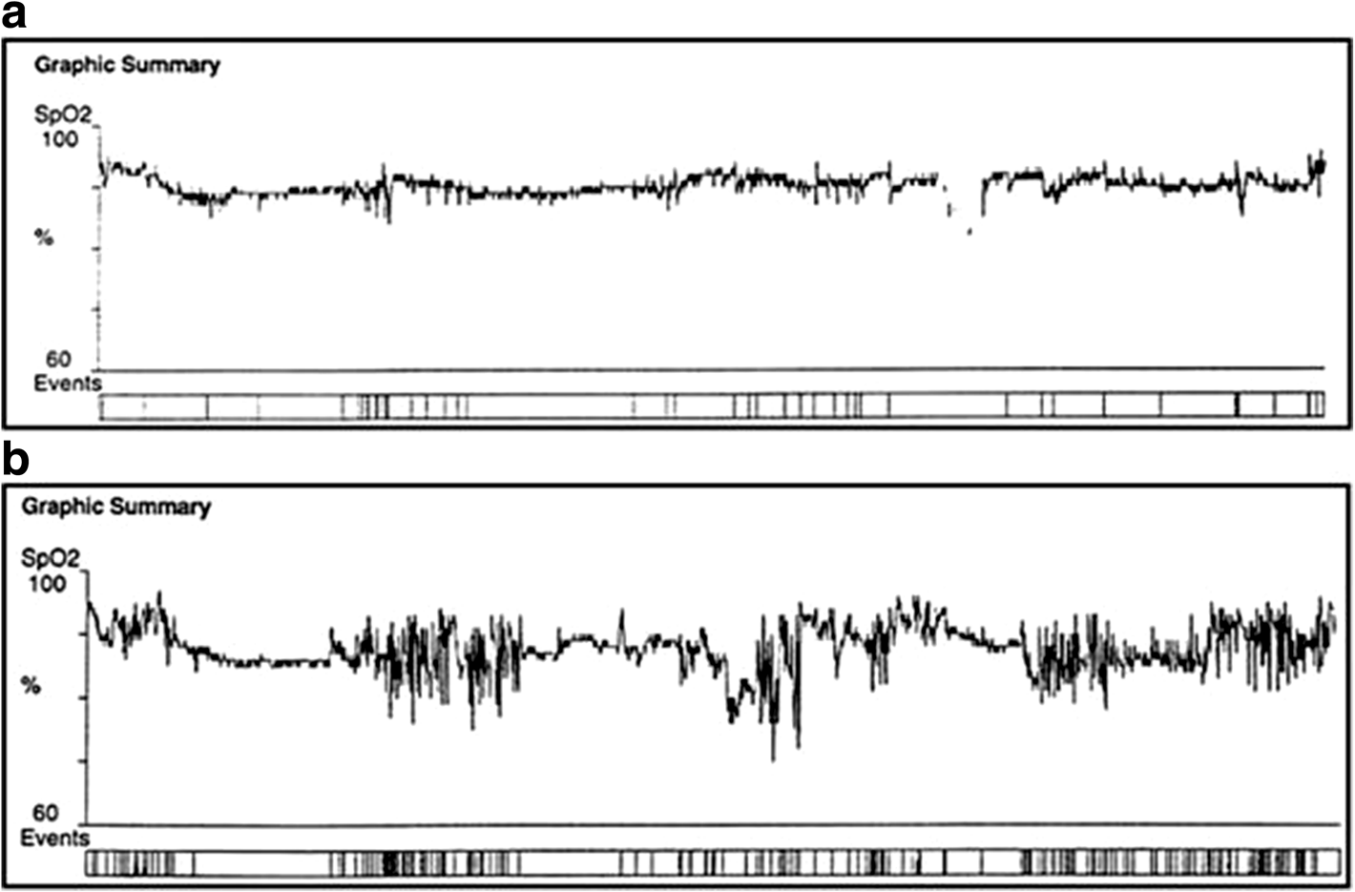
Nocturnal oximetry tracings in patients with COPD not qualifying for LTOT. **a** Significant nocturnal oxygen desaturation (>30% of the recording time with a saturation < 90%) without periodic variations in saturation, a tracing not suggestive of sleep apnea. **b** Significant nocturnal oxygen desaturation with cyclical changes in saturation suggesting sleep apnea, a tracing suggestive of sleep apnea
- nocturnal desaturation with suspicion of associated sleep apnea (i.e., cyclical changes in saturation in addition to the desaturations – Fig. 1b);
- no significant nocturnal desaturation (i.e., < 30% of the recording time with a saturation < 90%).
- patients with at least one abnormal recording demonstrating significant nocturnal desaturation with no suspicion of associated sleep apnea on both oximetries are directly eligible, without further testing.

**Fig. 2:**
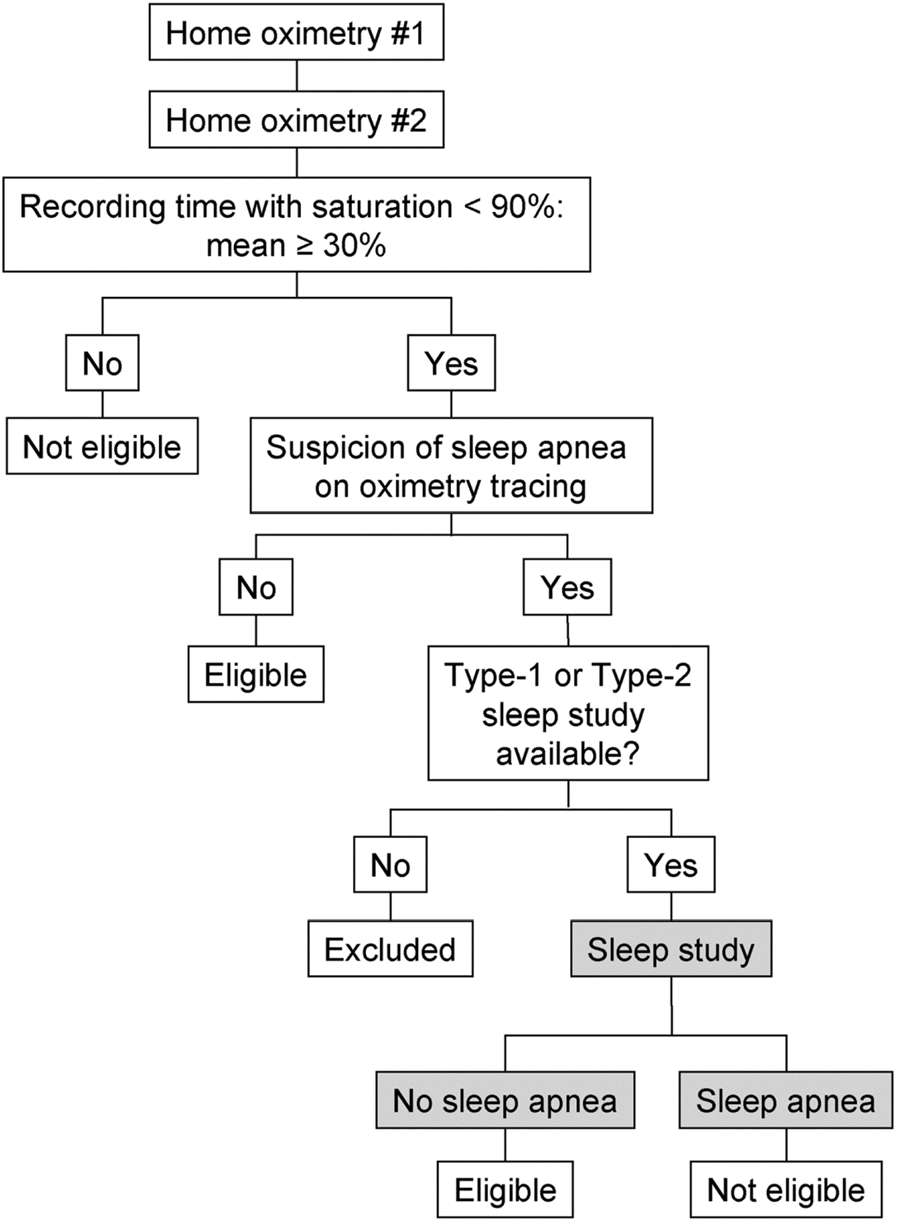
Diagnostic procedures. In case of oximetry tracing suggestive of sleep apnea, patients are excluded, unless sleep apnea is ruled out on the basis of a formal sleep study (either of Type-1 or Type-2 – shaded area) performed off-protocol

Significant “nocturnal desaturation” is defined on the home oximetry as ≥ 30% of the recording time (time in bed) with a transcutaneous arterial oxygen saturation < 90% [21, 25, 26]. Continuous nocturnal saturation (SaO_2_) monitoring is obtained with the PalmSAT 2500™ oximeter only (Nonin Medical Inc., Plymouth, MN, USA). Only recordings of at least 4-h duration are accepted. All patients undergo two oximetric studies [27] separated from each other by ≤ 2 weeks. Each oximetry recording is classified in one of three categories:

- significant nocturnal desaturation (i.e., ≥ 30% of the recording time with a transcutaneous arterial oxygen saturation < 90%) without suspicion of associated sleep apnea (i.e., steady tracing with non-periodic variation in saturation throughout sleep – Fig. 1a);

Patients with an oximetry tracing suggestive of sleep apnea are excluded, unless sleep apnea can be ruled out on the basis of a formal sleep study performed off-protocol. In such cases, the investigator must submit to the coordinating center the results of either a complete laboratory or full ambulatory polysomnography confirming the absence of sleep apnea before the patient is randomized [28]. Sleep apnea is defined as an apnea/hypopnea index ≥ 15 [24]. A flow diagram detailing the diagnostic procedures following the screening home oximetries is provided in Fig. 2.

### Intervention

Patients are randomly assigned to 3 years of treatment with either home NOT therapy or sham therapy with ambient air. Before randomization, patients are assessed and optimal pharmacological and non-pharmacological therapy is provided according to the clinician’s judgment. Because of the extended follow-up period, new therapies may emerge or ongoing trials may demonstrate positive effects of currently available treatment modalities on mortality. We therefore monitor and record co-interventions that arise throughout the trial period. Because clinical practice often varies across centers and new therapies are often introduced in different ways throughout centers, the randomization is stratified by centers.

#### Intervention arm

NOT is delivered overnight from an electrically-powered oxygen concentrator (NewLife Intensity Oxygen Concentrator, AirSep Corporation, Buffalo, NY, USA). The concentrator provides a constant source of oxygen from ambient air using a molecular sieve that removes nitrogen and water from air to deliver 95% oxygen at flow rates of up to 4 l/min. Patients are instructed to receive NOT throughout the night. The flow of oxygen is that allowing the nocturnal saturation to be > 90% for ≥ 90% of the recording time. This is assessed by the mean of pulse oximetry during a full-night recording (test night). Two liters of oxygen per minute are given during a first test night. If this flow of oxygen is not enough to keep the saturation > 90% for ≥ 90% of the recording time, then an additional test night is needed, with the oxygen flow rate increased by 1 l/min per night, up to 4 l/min.

#### Control arm

**Fig. 3:**
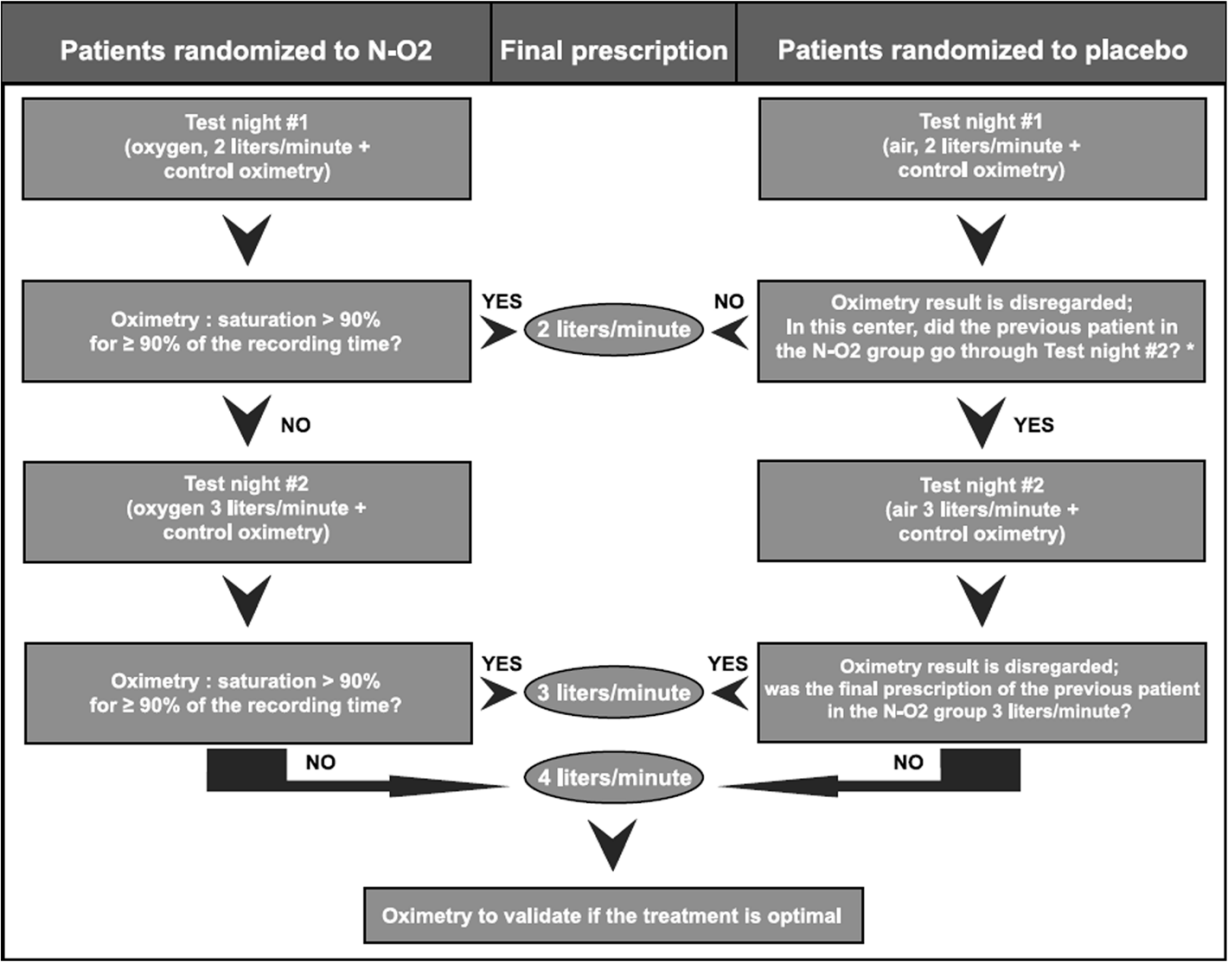
Nocturnal oxygen flow titration procedure

The patients allocated to the control group receive ambient air delivered overnight through the same concentrator rendered ineffective by bypassing the sieve beds. The sham concentrators have the same external appearance as the effective ones, allowing the trial to be double-blinded. We have received approval by Health Canada in order to proceed with the modifications on the oxygen concentrators; such approval by a regulatory agency was not mandatory in the European Community. The patients in the control group are also submitted to air flow adjustment (Fig. 3). To preserve blinding, patients in the control group are randomly submitted to additional test oximetries, with the airflow rate increased by 1 l/min, up to 4 l/min. The results of the oximetry performed during those nights are sent to the coordinating center but are disregarded.

#### Compliance issues

Objective daily duration of oxygen therapy (or placebo) is measured using the concentrators’ counter clock recording the number of hours of utilization. This information is recorded during regular home visits scheduled every 4 months. Patient receiving oxygen or sham therapy during at least 70% of the total time in bed over the 3-year trial are considered as compliant. Total time in bed is estimated from the typical daily time in bed that is self-reported at baseline.

### Outcomes

#### Primary outcome

The primary outcome of the INOX trial is a composite of “all-cause mortality” or “requirement for LTOT”. All-cause mortality is preferred over disease-specific mortality because of difficulties in classifying causes of death [29] and the lack of validity of death certificates in patients with COPD [30]. The widely accepted criteria for LTOT derived from the Nocturnal Oxygen Therapy Trial [3] are used to define “requirement of LTOT”. These criteria are met in either of the two following clinical circumstances:

- In stable patients:
  Patients may become severely hypoxemic over time due to progressive deterioration of the disease that characterizes its natural course. In such circumstances, the requirement for LTOT is captured through periodic surveillance. At each protocol-based follow-up visit, patients are submitted to pulse oximetry at rest. If pulse oximetry at rest gives a saturation ≥ 92%, then direct arterial blood gas measurement is not required. Otherwise, arterial blood gas must be sampled for direct PaO2 measurement. Patients whose PaO2 falls below 56 mmHg during the follow-up period are offered conventional LTOT [3]. The trial end-point is then reached.

- In unstable patients:
  Patients may become severely but temporarily hypoxemic during an acute exacerbation of COPD necessitating hospitalization. In such circumstances, short-term oxygen therapy may be prescribed for a short period of time, especially if oxygen therapy allows the patient to be safely discharged from the hospital [31]. Any decision regarding the maintenance of oxygen therapy (i.e., the requirement of LTOT following short-term oxygen therapy) must be made after a period of clinical stability of at least 30 days [31]. Reevaluation must occur within 12 weeks after the end of the treatment of the exacerbation. The study primary endpoint is reached only when LTOT criteria are met.

#### Secondary outcomes

Disease-specific quality of life (St. George’s Respiratory Questionnaire [32–34]), generic quality of life (SF-36 [35, 36]) and associated utility scores (SF-6D [37]), costs and health care utilization are secondary outcomes. The estimates of the cost of COPD treatment for the two intervention groups will be based on the utilization of the intervention resources during the study period. The social and health care perspectives will be adopted. Professional time, intervention materials and health care utilization will be considered.

### Sample size

In their randomized trial, the French group reported a 3-year mortality of 20% [21] and a rate of LTOT prescription of 29%, with 40% of the study population reaching one or the other of the endpoints. These figures are consistent with the survival rate of patients with COPD reported in the literature [38], including a large North American study (*n* = 985) [39]. Therefore, we anticipate the 3-year event rate (i.e., mortality or requirement for LTOT) among patients not receiving NOT to be around 40%. We targeted a 30% relative reduction in this composite outcome in the experimental group (i.e., an event rate in the control group of 40% and an event rate of 28% in the NOT group, or an absolute difference in event rates of 12%). This absolute difference is consistent with the minimal clinically important difference elicited by Canadian pulmonologists in a national survey that we conducted prior to the INOX trial [26]. The level of statistical significance is set at *p* = 0.05 (two-sided). Translating this in terms of our proposed log rank test, we calculated that 300 patients per group (total sample size: 600) will provide us with a power of 90% [40].

### Recruitment

In a feasibility study, we demonstrated that 40% of the patients with moderate-to-severe COPD not qualifying for continuous oxygen are nocturnal desaturators [10]. In our survey of Canadian pulmonologists, we found that, on average, 30% of the respondents’ practice (including that of our co-investigators) is dedicated to the care of patients with COPD [26]. This information clearly demonstrates that nocturnal oxygen desaturation in patients with COPD is not a rare occurrence and that the investigators have access to a large pool of potentially eligible patients.

### Participant timeline

Time schedule of assessments and follow-up visits for participants is reported in the Table 1.

**Table 1:** Schedule of follow-up procedures, including a fourth year of follow-up according to the Steering Committee’s 2012 recommendation

	Time line (months)												
	0	4	8	12	16	20	24	28	32	36	40	44	48
Consent form	√												
Baseline/follow-up general health information	√			√			√			√			√
Nocturnal oxymetry	√												
Pulse oxymetry		√	√		√	√		√	√	√	√	√	√
Arterial blood gas	√	√^a^	√^a^	√^a^	√^a^	√^a^	√^a^	√^a^	√^a^	√^a^	√^a^	√^a^	√
Pulmonary function tests (spirometry, lung volumes and DLCO)	√			√			√			√			√
Quality-of-life questionnaires	√			√			√			√			√
Health Care questionnaire (follow-up call or visit)^b^		√	√	√	√	√	√	√	√	√	√	√	√
Home visits for compliance^c^		√	√	√	√	√	√	√	√	√	√	√	√

^a^Depending on the result of the arterial saturation in oxygen measured by pulse oximetry

^b^Health care utilization is measured through telephone contacts with patients every 2 months

^c^Home visits are performed by home care service provider staff

### Allocation

The randomization process consists of a computer-generated random listing of the two treatment allocations blocked by variable blocks of four and six in alternance and stratified by site. Randomization is through central allocation and coordinated by the *Laboratoire de télématique biomédicale* (LTB) of the Respiratory Health Network of the *Fonds de recherche du Québec – Santé (FRQS)*. Physicians and research staff are unaware of the treatment allocation prior to or following randomization. At the time of randomization, each patient is provided with a site-specific study number according to the randomization schedule. The results of the randomization is only communicated by the LTB to the individual responsible for the preparation, delivery and installation of the home concentrators and oxygen flow titration.

### Data collection and management

Standardized case report forms have been developed specifically for the trial. Completed forms are periodically sent by the participating centers to the Coordinating centre for verification and data entry though a secured website using range checks for data values. The stored data is secured at the LTB. Clinical centres are reimbursed only after complete data is transmitted to the Coordinating center.

### Data monitoring

An independent Data and Safety Monitoring Board (DSMB) assists and advises the Steering Committee to protect the validity and credibility of the trial. The DSMB operates according to the terms of a charter that was developed according to the DAMOCLES Study Group’s recommendations [41]. The DSMB receives and reviews annually the progress and accruing data of the trial and provides advice on the conduct of the trial to the Steering Committee. The DSMB may request the conduct of an interim analysis.

### Statistical analysis

#### Primary analyses

The primary analysis will follow an intent-to-treat approach. The distribution of time to achievement of the primary composite outcome (all-cause mortality or requirement for LTOT) will be estimated by the Kaplan-Meier method, and the difference between the two study groups will be evaluated with a log-rank test. The estimated relative risk of mortality or requirement for LTOT with its 95% confidence interval will be computed. Multivariable analyses with the Cox proportional-hazards model will be used to estimate the simultaneous effects of prognostic factors (including gender, age, FEV1, and comorbidities) and on the composite outcome. Differences will be considered to be statistically significant at the 0.05 level (two-sided).

#### Subgroup analyses

The effect of nocturnal oxygen may depend on the severity of nocturnal desaturation which may be defined in terms of % of time in bed with a saturation < 90% or in terms of mean saturation throughout the recording time. Accordingly, in addition to the traditional threshold of 30% of the time with a saturation < 90%, the effect of nocturnal oxygen will be analyzed according to various thresholds of desaturation.

#### Cost effectiveness analysis

An incremental cost effectiveness analysis will be undertaken to assess the efficiency of NOT. The overall costs and effects of the two groups will be used to calculate incremental cost effectiveness ratios according to the following equation: R = (C_T_ − C_C_)/(E_T_ − E_C_) = ΔC/ΔE, where R is the incremental cost effectiveness ratio, C_C_ and E_C_ are the means of the control group costs and effect, respectively, C_T_ and E_T_ are the means of the treatment group costs and effect, respectively, and ΔC and ΔE are the incremental cost and incremental effect, respectively [42]. Protocol-specific costs will be disregarded in the control group. The effect of therapy will be defined in terms of mortality, life-years and utility.

## Discussion

The INOX protocol development, funding and implementation result from a series of international consultations with trialists and expert clinicians in the area of COPD. Although its methodology is straightforward, debates took place in the early stages of the study regarding important aspects of the trial including (1) the need for blinding, (2) its composite outcome, and (3) the exclusion of sleep apnea.

### Need for blinding

Sham concentrators are more expensive than effective ones. Obtaining permission from the Canadian regulatory agency to produce such equipment added to the complexity of the trial implementation. Therefore, the first area of discussion was about the need of placebo in a trial whose primary outcome includes mortality. However, mortality is not the only endpoint. The primary outcome is also composed of the requirement for LTOT which is determined at least in part by the actions of clinicians. Although it follows strict criteria and guidelines defined in this protocol, there is conceivably potential for more aggressive surveillance (monitoring) of arterial blood gases in those in the control group, leading to an increased likelihood of prescription of LTOT in this group. In this regard, INOX could not be conducted other than as a double-blind, placebo-controlled trial.

### Composite outcome

Although we realize the difficulties related to composite outcomes in clinical trials [43, 44], the requirement of LTOT must represent an endpoint of this trial for clinical and methodological reasons. The primary reason is that the condition of participants may deteriorate to the point that LTOT is required. This situation is particularly problematic because LTOT compulsorily includes sleep time (and therefore NOT). If mortality was the only outcome, and if LTOT was prescribed because of disease progression to a patient allocated to NOT, NOT would then become LTOT (which is of proven effectiveness in improving survival in COPD). Similarly, if LTOT was prescribed in a patient allocated to the control group, it would then represent an important contamination. Both situations would represent important threats to the validity of our trial.

We understand that the choice of a composite outcome requires that its components (1) are of similar importance, (2) occur with similar frequency [43]. In order to support our view that our composite outcome is appropriate, we first derived utility scores (SF-6D scores) [37] in 102 patients with oxygen-dependent COPD. The mean utility score was 0.60 (SD: 0.11) [45]. For comparison, this utility score is worse than that attached to a large myocardial infarction, stroke leaving permanent moderate deficit, or dissecting or ruptured aortic aneurysm [46], three conditions considered in the cardiovascular literature as appropriate in composite outcomes that include mortality [44]. Regarding the relative frequency of the two components of the composite outcome, we have already made the point that mortality and requirement for LTOT should occur with similar incidence during the trial (see Sample size, above).

### Exclusion of sleep apnea

COPD and obstructive sleep apnea (OSA) are common conditions. The combination of COPD and sleep apnea is referred to as the “overlap syndrome” [47]. A population-based study indicated that both conditions are not linked by common pathophysiological mechanisms, and that their association is only by chance [48]. The routine utilization of sleep studies in patients with COPD to distinguish between sleep apnea and nocturnal oxygen desaturation alone (i.e. without sleep apnea) is controversial. On one hand, the access to diagnostic facilities for patients with suspected sleep apnea in Canada and many other jurisdictions is unfortunately very limited [49, 50], and the requirement of a polysomnography for all patients in the frame of this study would be unrealistic. On the other hand, 42% of the Canadian pulmonologists think that all COPD patients with significant nocturnal desaturation should have a polysomnography to rule out sleep apnea [26]. In a blind comparison of home nocturnal oximetry and laboratory polysomnography in consecutive patients with COPD and nocturnal oxygen desaturation, we found that, in patients with significant nocturnal oxygen desaturation, home nocturnal oximetry has high negative predictive value for the diagnosis of OSA (unpublished data). However, home nocturnal oximetry has a poor positive predictive value for the diagnosis of OSA [51]. It is on the basis of this study that we constructed the algorithm for the patients’ screening and selection (Fig. 2).

### Important protocol modification

Despite our continuing efforts to increase the patients’ accrual rate across participating centers, recruitment has been a challenge since the beginning of the trial in November 2009. It has become obvious that the target sample size of 600 patients would not be reached within a reasonable period of time. In order to increase the number of events, the Steering Committee strongly recommended in 2012 that the follow-up period be extended from 3 to 4 years. The rationale to initially propose a 3-year trial was from the results of the British trial [4], in which 500 days elapsed before any effect of continuous oxygen therapy appeared, when compared to no oxygen therapy at all. By extending the follow-up period to 4 years, we anticipate that 50% of those allocated in the control group will reach the primary outcome. Accordingly, aiming at a 30% relative reduction in event rate in the experimental group (i.e., an event rate in the control group of 50% and an event rate of 35% in the NOT group, or an absolute difference in event rates of 15%), we calculated that 160 patients per group would provide us with a power of 80% (type-1 error: 0.05, two-sided). During this fourth year of follow-up, the same procedures as in Year 3 apply.

### Implication for practice

Why should clinicians be interested in nocturnal oxygen desaturation? COPD clearly represents a significant burden of health care systems wherever it has been assessed [52]. Home oxygen therapy comes in second place (only after hospitalizations) among the most expensive health care resources for COPD. In the Canadian cohort of the Confronting COPD Survey (3265 individuals; mean age: 63 years; 44% female), outpatient treatment for COPD accounted for over 30% of total direct costs, the majority of which was for home oxygen therapy. Overall, home oxygen therapy accounted for almost 20% of the entire annual direct costs for COPD [53]. Informal surveys among respiratory home care programs in the province of Quebec (Canada) indicate that 15–20% of those who receive home oxygen therapy through these programs have been prescribed oxygen for nocturnal utilization only. A more formal survey of Canadian pulmonologists revealed that 87% of them had already prescribed nocturnal oxygen in COPD [26]. Given the resources allocated to nocturnal oxygen therapy, its prescription should therefore be justifiable by demonstrating an improvement in clinical outcomes other than the mere correction of nocturnal oxygen desaturation.

In the most recent COPD international guidelines [54, 55], the issue of nocturnal oxygen therapy is not addressed. Two workshops of the National Heart, Lung, and Blood Institute identified nocturnal oxygen therapy as a research priority in COPD [56, 57]. This situation stimulated our planning and implementation of the INOX trial.

## Abbreviations

COPD: Chronic obstructive pulmonary disease
ECG: Electrocardiogram
FEV1: Forced expiratory volume in 1 s
FEV1/FVC: Forced expiratory volume in 1 s to forced vital capacity ratio
INOX: International Nocturnal Oxygen
LTB: Laboratoire de télématique biomédicale
LTOT: Long term oxygen therapy
NOT: Nocturnal oxygen therapy
OSA: Obstructive sleep apnea
SF-36: Medical Outcome Survey - Short Form 36
